# Genetic variants in miR-145 gene are associated with the risk of asthma in Taiwan

**DOI:** 10.1038/s41598-022-18587-w

**Published:** 2022-09-07

**Authors:** Shou-Cheng Wang, Chia-Wen Tsai, Wen-Shin Chang, Ning-Yi Hsia, Mei-Chin Mong, Yun-Chi Wang, Te-Chun Hsia, Jian Gu, Da-Tian Bau

**Affiliations:** 1 Taichung Armed Forces General Hospital, Taichung, Taiwan, ROC; 2 National Defense Medical Center, Taipei, Taiwan, ROC; 3 Graduate Institute of Biomedical Sciences, China Medical University, Taichung, Taiwan, ROC; 4 Terry Fox Cancer Research Laboratory, Department of Medical Research, China Medical University Hospital, 2 Yuh-Der Road, Taichung, 404 Taiwan, ROC; 5 Department of Epidemiology, The University of Texas MD Anderson Cancer Center, Houston, TX 77030 USA; 6 Department of Food Nutrition and Health Biotechnology, Asia University, Taichung, Taiwan, ROC; 7 The Ph.D. Program for Health Science and Industry, China Medical University, Taichung, Taiwan, ROC; 8 Department of Bioinformatics and Medical Engineering, Asia University, Taichung, Taiwan, ROC

**Keywords:** Molecular biology, Biomarkers, Medical research, Molecular medicine

## Abstract

Asthma is a chronic airway inflammation disease and the diagnosis and treatment strategies remain difficult. MicroRNAs play important roles in many biological and pathological processes including asthma development. There is no study confirming the contribution of genetic variants in *miR-145* to asthma etiology. We hypothesize that single nucleotide polymorphisms (SNPs) in the promoter region of *miR-145* may be associated with the risk of asthma in Taiwanese. We used a case–control study to test this hypothesis. In 198 asthma patients and 453 healthy controls, the genotypes of *miR-145* rs4705342 and rs4705343 were determined, and the associations of *miR-145* genotypes with asthma risk and severity were evaluated. The distribution of *miR-145* rs4705342 genotypes between asthma patients and non-asthmatic control groups were significantly different (*p* = 0.0187). In multivariable logistic regression analysis, compared with the wild-type TT genotype, individuals carrying the variant genotypes had progressively decreased risks of asthma: the odds ratio (OR) for the heterogeneous variant genotype (CT) and homozygous variant genotype (CC) was 0.77 (95% CI 0.55–1.10, p = 0.1788) and 0.41 (95% CI 0.21–0.79, p = 0.0102), respectively (p for trend = 0.0187). In allelic test, the C allele was associated with a 31% reduced risk of asthma (OR = 0.69, 95% CI 0.53–0.90, *p* = 0.0070). In addition, the rs4705342 variant genotypes were correlated with the symptom severity (*p* = 3 × 10^–5^). Furthermore, the variant genotypes correlated with lower miR-145-5p expression level in serum (*p* = 0.0001). As for rs4705343, there was no differential distribution of genotypes between cases and controls. Our data provide evidence for *miR-145* rs4705342 to serve as a novel biomarker for asthma risk prediction.

## Introduction

Asthma is a prevalent chronic obstructive disease characterized by the remodeling of airways. Globally, about 300 million people are attacked by asthma, and its prevalence is continuously increasing^1,2^. The incidence of asthma varies among different areas in the world. Developed countries generally have a significantly higher incidence of asthma than developing countries due to higher environmental exposures such as smog and air particles^3^. Previous studies have found that the occurrence of asthma has a strong genetic component, with a heritability of up to 60–80%^4–6^. In 2014, an animal model of human asthma suggested that about two hundred genes may contribute to the etiology of asthma^7^. There have been numerous candidate gene studies investigating the contributions of DNA repair genes, extracellular metabolism genes, cell cycle regulating genes, cytokines and immunological genes to asthma susceptibility, focusing on the gene-environment interactions and endotypes of asthma etiology^6,8–13^. Additional genetic susceptibility loci for asthma remain to be identified.

MicroRNAs (miRNAs) are small non-coding RNAs that act as regulators of gene expression as they bind to the 3’-untranslated regions of their target mRNAs and can influence many cellular signaling networks^14^. MiRNA gene SNPs can affect several processes including primary target gene transcription, pri-/pre-miRNA processing, or miRNA-mRNA interactions^15^. MiRNA dysregulation has been associated with various diseases, especially cancer, since miRNA can target genes involved in regulation of cell proliferation and survival, DNA repair and immune response^16^.

MiR-145-5p was previously found to be significantly increased in the plasma of patients with chronic obstructive pulmonary disease and asthma, indicating that plasma miR-145-5p is a specific biomarker of respiratory disease^17^. Ozone, a poisonous form of oxygen, is associated with lots of adverse health effects and significantly increased the expression of a variety of miRNAs, including miR-145 in human bronchial airways^18^. Moreover, in house dust mite (HDM)-induced asthma mice models, HDM increased the expression of miR-145-5p, while miR-145-5p inhibition reduced eosinophilic inflammation, mucus hypersecretion, type 2 helper T cell (Th2) cytokine production, and airway hyperresponsiveness^19^. Furthermore, Liu and his colleagues have found that miR-145-5p was aberrantly overexpressed in airway smooth muscle cells exposed to cytokine stimulation that mimic the etiology of asthma patients^20^. However, although the upregulation of miR-145-5p plays a role in the pathogenesis of asthma, its underlying mechanism remains unclear.

*MiR-145* gene is located in the extremely conserved chromosomal region 5q32^21^. In 2013, several SNPs were identified upstream from the transcription start site of *miR-145*^21^. Among them, rs4705342 and rs4705343 were reported to be functional, with C allele carriers exhibiting relatively higher reporter gene activity by increasing the extent of NF-κB binding^22–24^. Although more and more evidence has suggested that *miR-145* gene SNPs were associated with cancer risks^25,26^, the influence of *miR-145* genotypes on asthma risk has never been reported. In 2021, Tiwari and colleagues reported that the expression of miR-145-5p is associated with the early decline patterns of lung function growth leading to chronic obstructive pulmonary disease (COPD) in children with asthma and additionally increases airway smooth muscle cell proliferation^27^. It adds indirect biological evidence that *miR-145* genotypes may play a role in asthma etiology. In this study, we first examine the associations of *miR-145* rs4705342 and rs4705343 genotypes with the risk and severity of asthma, then reveal the genotype–phenotype correlation between *miR-145* genotypes and serum miR-145-5p expression level.

## Methods

### Recruiting asthmatic cases and non-asthmatic healthy controls in Taiwan

One hundred and ninety-eight asthmatic cases were recruited at China Medical University Hospital in central Taiwan. Simultaneously, 453 non-asthmatic individuals matched by gender and age were enrolled as controls^10^. The study was approved and supervised by Research Ethics Committee of China Medical University Hospital (CMUH106-REC1-004), and performed in accordance with the Declaration of Helsinki. The symptom severity for asthma was verified by at least two experienced pulmonary physicians according to the Global Initiative for Asthma guidelines^2^. Specifically, the patients are separated into 4 groups based on the level of treatment required to control the symptoms and exacerbations: treated with as-needed inhaled corticosteroid (ICS)-formoterol alone (group 1, mildest), with low-intensity maintenance controller treatment of ICS-formoterol, leukotriene receptor antagonists or chromones (group 2), with low dose ICS-long acting beta2 agonist (LABA) (group 3), and with high dose ICS-LABA (group 4, severest)^2^.

### Genotyping

Peripheral blood was collected from all subjects, and their genomic DNA was extracted^28^. Genotyping methods were described previously^24^. Briefly, the rs4705342 genotype was determined by a TaqMan Assay on an ABI 7500 Real-Time PCR System (Applied Biosystems, Foster City, CA, USA), and the rs4705343 genotype was identified via the polymerase chain reaction-restriction fragment length polymorphism methodology.

### Measuring serum miR-145-5p expression

Total RNA was extracted from 45 serum samples using Trizol Reagent (Invitrogen, Carlsbad, CA, USA). The expression levels of miR-145-5p were measured by real-time quantitative reverse transcription-PCR on FTC-3000 real-time quantitative PCR instrument (Funglyn Biotech Inc., Canada)^29–31^. The levels of miR-145-5p were normalized to the levels of GAPDH expression and compared with each other. The TT wild-types of *miR-145* rs4705342 and rs4705343 were set as 1.0 as reference. Each sample was measured three times.

### Statistical analysis

The frequencies of rs4705342 and rs4705343 of the control group were estimated by good-of-fit Chi-square test, examining for fitness of Hardy–Weinberg equilibrium. The *Student’s t*-test was used to examine the differential distributions of ages between the case and control groups. The Pearson’s Chi-square test was used to examine the differential distribution of various genotypes and the interaction between genotypes with symptom severity. Multivariable logistic regression analysis was used to estimate the adjusted odds ratios (aORs) and 95% confidence intervals (CIs) adjusting for age, gender and smoking behavior. Any *p*-value less than 0.05 is considered as statistically significant.

### Ethics approval and consent to participate

The Research Ethics Committee of China Medical University Hospital approved the study protocol (CMUH106-REC1-004) and waived the need for informed consent due to the study design.

## Results

### Demographics of asthmatic and non-asthmatic groups

The 198 asthmatic cases and 453 no-asthmatic controls were frequency-matched on age and gender. There was no significant difference in smoking behavior between the cases and controls (*p* = 0.7161). For pulmonary functions, both the average ratio of forced expiratory volume in the first second (FEV1) to forced vital capacity (FVC) (FEV1/FVC, %) and the percentage of predicted FEV1 (FEV1%), were lower among the asthmatic cases than among the control subjects (both *p* < 0.0001). There were 60 (30.3%), 65 (32.8%), 34 (17.2%) and 39 (19.7%) patients belonging to the symptom severity group 1 (mild), 2, 3 and 4 (severe), respectively (Table 1).

**Table 1:** Distributions of baseline characteristics among the 198 asthmatic patients and 453 controls.

Index	Controls (n = 453)		Cases (n = 198)		*p*-value*
	n	%	n	%	
**Age (years)**					
25–40	285	63.4%	133	67.2%	
> 40	168	36.6%	65	32.8%	0.2972
**Gender**					
Male	190	41.9%	83	41.9%	
Female	263	58.1%	115	58.1%	0.9956
**Smoking status**					
Never	326	72.0%	139	70.2%	
Ever	127	28.0%	59	29.8%	0.7161
**Pulmonary functions (mean ± SD)**					
FEV1/FVC (%)	80.8 ± 8.1		62.0 ± 13.0		< 0.0001
FEV1%	92.9 ± 5.8		69.1 ± 12.9		< 0.0001
**Symptoms severity**					
1 (mild)			60	30.3%	
2			65	32.8%	
3			34	17.2%	
4 (severe)			39	19.7%	

*FEV1* forced expiratory volume in first second, *FVC* forced vital capacity, *FEV1%* percent of predicted FEV1.

*Chi-square without Yate’s correction test or *Student’s* t-test.

### Association of *miR-145* genotypes with the risk of asthma

First, the genotypic frequencies of rs4705342 in the control group fit well with the Hardy–Weinberg equilibrium (*p* = 0.4451, Table 2). Second, the genotypic frequencies of rs4705342 were differentially distributed among the asthmatic cases and the non-asthmatic healthy controls (*p* for trend = 0.0187). In multivariable logistic regression analysis adjusting for age, gender and smoking behavior, compared with the wild-type TT genotype, individuals carrying the variant genotypes had progressively decreased risks of asthma: the ORs for the heterogeneous variant genotype (CT) and homozygous variant genotype (CC) were 0.77 (95% CI 0.55–1.10, *p* = 0.1788) and 0.41 (95% CI 0.21–0.79, *p* = 0.0102), respectively (*p* for trend = 0.0187) (Table 2). In the dominant model, individuals with the variant genotypes (CT + CC) exhibited a 31% reduced risk of asthma (OR = 0.69, 95% CI 0.50–0.97, *p* = 0.0385) (Table 2).

**Table 2:** Distributions of rs4705342 genotypic frequencies between asthmatic patient and control groups.

Polymorphism	Genotype	Cases	Controls	*p*-Value	OR (95%CI)	Adjusted OR (95%CI)^a^
rs4705342	TT	106 (53.5%)	201 (44.4%)		1.00 (Ref)	1.00 (Ref)
	CT	80 (40.4%)	196 (43.3%)	0.1788	0.77 (0.55–1.10)	0.79 (0.63–1.14)
	CC	12 (6.1%)	56 (12.3%)	0.0102*	**0.41 (0.21–0.79)**	**0.48 (0.25–0.77)**
*P*_trend_				0.0187*		
*P*_*HWE*_				0.4451		
Recessive	TT + CT	186 (93.9%)	397 (87.6%)		1.00 (Ref)	1.00 (Ref)
	CC	12 (6.1%)	56 (12.3%)	0.0227*	**0.46 (0.24–0.87)**	**0.51 (0.27–0.84)**
Dominant	TT	106 (53.5%)	201 (44.4%)		1.00 (Ref)	1.00 (Ref)
	CC + CT	92 (46.5%)	252 (55.6%)	0.0385*	**0.69 (0.50–0.97)**	**0.71 (0.54–0.96)**

*p-*values were calculated by Chi-square without Yates' correction.

*P*_*trend*_* p*-value for trend, *P*_*HWE*_* p*-value for Hardy–Weinberg equilibrium.

*p < 0.05.

^a^Adjusted for age, gender, and smoking status.

The rs4705343 genotypes were not significantly associated with the risk of asthma in any models (Table 3).

**Table 3:** Distributions of rs4705343 genotypic frequencies between asthmatic patient and control groups.

Polymorphism	Genotype	Cases	Controls	*p*-Value	OR (95%CI)	Adjusted OR (95%CI)*
rs4705343	TT	86 (43.4%)	213 (47.0%)		1.00 (Ref)	1.00 (Ref)
	CT	95 (48.0%)	199 (43.9%)	0.3956	1.18 (0.83–1.68)	1.21 (0.85–1.59)
	CC	17 (8.6%)	41 (9.1%)	0.9328	1.03 (0.55–1.91)	1.06 (0.58–1.88)
*P*_trend_				0.6314		
*P*_*HWE*_				0.5715		
Recessive	TT + CT	181 (91.4%)	412 (90.9%)		1.00 (Ref)	1.00 (Ref)
	CC	17 (8.6%)	41 (9.1%)	0.9665	0.94 (0.52–1.71)	0.96 (0.57–1.78)
Dominant	TT	86 (43.4%)	213 (47.0%)		1.00 (Ref)	1.00 (Ref)
	CC + CT	112 (56.6%)	240 (53.0%)	0.4478	1.16 (0.83–1.62)	1.11 (0.84–1.57)

*p-*values were calculated by Chi-square without Yates' correction.

*P*_*trend*_* p*-value for trend, *P*_*HWE*_* p*-value for Hardy–Weinberg equilibrium.

*Adjusted for age, gender, and smoking status.

### Allelic frequency distribution analysis

The allelic frequency analysis showed that individuals with the C allele at rs4705342 were at a significantly lower risk of asthma than those with the T allele (OR = 0.69, 95% CI 0.53–0.90, *p* = 0.0070) (Table 4). The rs4705343 alleles were not significantly associated with asthma risk (Table 4).

**Table 4:** Distribution of *miR-145* allelic frequencies among asthmatic patients and non-asthmatic controls.

Allelic type	Asthmatic cases, n (%)	Non-asthmatic controls, n (%)	OR (95%CI)	*p-*value*
**rs4705342**				
Allele T	292 (73.7)	598 (66.0)	1.00 (Reference)	
Allele C	104 (26.3)	308 (34.0)	**0.69 (0.53–0.90)**	**0.0070**^**#**^
**rs4705343**				
Allele T	267 (67.4)	625 (69.0)	1.00 (Reference)	
Allele C	129 (32.6)	281 (31.0)	1.07 (0.83–1.38)	0.6222

*OR* odds ratio, *CI* confidence interval.

*Based on Chi-square test without Yates’ correction.

^**#**^Statistically significant.

### *MiR-145* rs4705342 genotypes were associated with symptom severity

We are interested in whether the rs4705342 genotypes are associated with symptom severity. To answer this question, the asthmatic cases were stratified according to their rs4705342 genotypes and symptom severity. The asthma patients with CT and CC genotypes were pulled together. The results showed that variant genotype (CT or CC) carriers were at a lower risk to suffer from severe symptom than those wild-type (TT) ones (*p* = 3 × 10^–5^) (Table 5). There were no significant associations between rs4705343 genotypes and symptom severity (Table 5).

**Table 5:** Association of *miR-145* SNPs with the symptoms severity among asthmatic patients.

Genotype	Symptom severity, n (%)				*p-*value*
	1 (mildest)	2	3	4 (severest)	
**rs4705342**					
Wild-type genotype	24 (22.6)	27 (25.5)	23 (21.7)	32 (30.2)	
Variant genotypes	36 (39.1)	38 (41.3)	11 (12.0)	7 (7.6)	**3.0 × 10**^**–5#**^
**rs4705343**					
Wild-type genotype	26 (30.2)	32 (37.2)	13 (15.1)	15 (17.5)	
Variant genotypes	34 (30.3)	33 (29.5)	21 (18.8)	24 (21.4)	0.6468

*Chi-square without Yate’s correction test.

^**#**^Statistically significant.

### Correlation of genotypes at rs4705342 with serum levels of miR-145-5p

**Figure 1:**
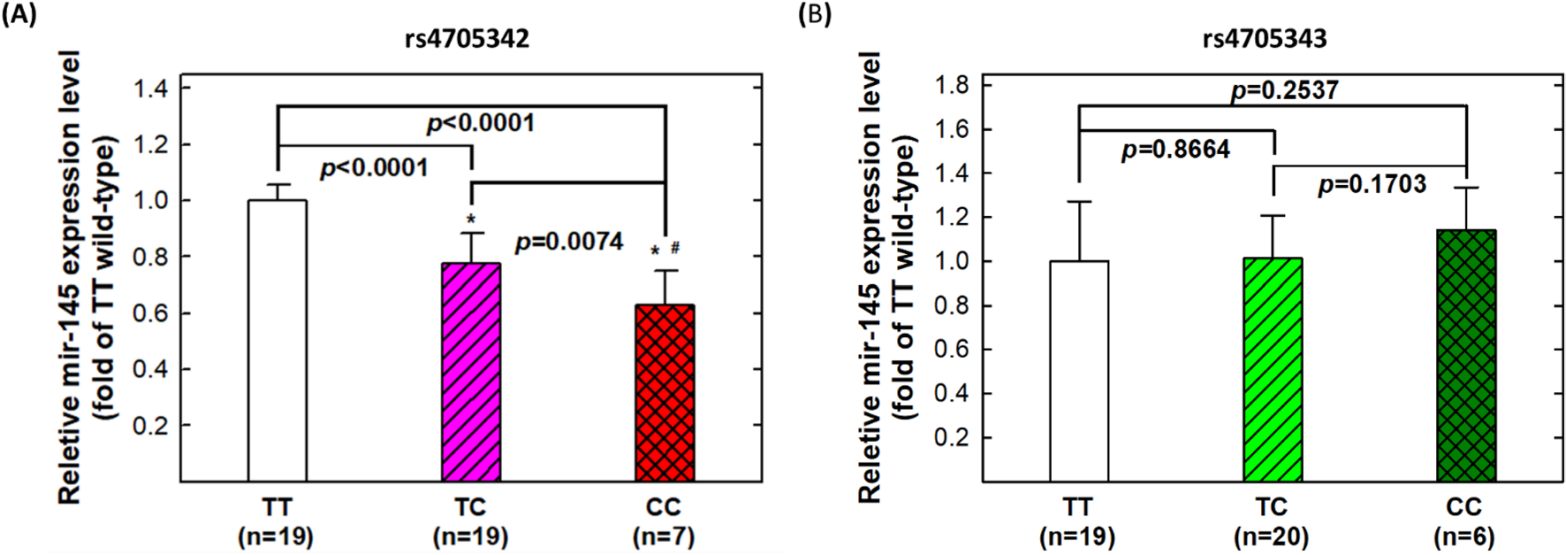
Correlation between rs4705342 and rs4705343 genotypes and miR-145-5p expression in the serum of non-asthmatic healthy subjects. (**A**) MiR-145-5p expression levels of 45 healthy samples according to rs4705342 genotypes; (**B**) MiR-145-5p expression levels of 45 healthy samples according to rs4705343 genotypes. *Statistically significantly different from TT genotypes; ^#^statistically significantly different from TC genotypes.

We then evaluated the correlation of various genotypes of *miR-145* rs4705342 and rs4705343 with serum miR-145-5p level in 45 healthy controls. The results showed that the rs4705342 variant genotypes (CC and CT) were associated with progressively reduced serum miR-145-5p levels than the wild-type genotype (TT): the serum miR-145-5p levels of CT (0.7742) and CC (0.5429) genotype carriers were 23% and 46% lower than that of the wild-type TT genotype carriers (*p* < 0.0001 for both comparison) (Fig. 1A). The comparison of homozygous variant genotype (CC) with heterogeneous variant genotype (CT) carriers was also significantly different (0.5429 versus 0.7742, *p* = 0.0074) (Fig. 1A). There was no significant difference of the expression levels of miR-145-5p for the rs4705343 TT, CT or CC genotype carriers (all *p* > 0.05) (Fig. 1B).

## Discussion

Asthma, a chronic and allergic respiratory illness, is caused by the combination of internal and external factors, while miRNAs may serve as critical internal factors^20,32^. MiR-145-5p, together with miR-138, miR-214, miR-371 and miR-544, can modulate the balance of T helper cells in asthma etiology^32^. MiR-145-5p has been suggested as a suppressor of tumorigenesis, although its expression levels were not conclusive in certain types of cancer. Most importantly, miR-145-5p has been found to be significantly up-regulated in asthma^32^. In that study, among 30 asthmatic patients and 25 healthy subjects, serum miR-145-5p level was found to be significantly higher in asthma patients than in healthy subjects^32^. In addition, its over-expression can suppress the expression of Runx3 and regulate the balance between Th1 and Th2^32,33^. To our surprise, there was no report on either the association of *miR-145* genotypes with asthma risk or asthma severity.

In the current study, we revealed that the genotypic proportions for TT, CT, and CC of rs4705342 were 44.4, 43.3, and 12.3% in the control Taiwanese population (Table 2). For the first time, the variant genotypes (CT and CC) and the C variant allele of rs4705342 were found to be significantly associated with reduced asthma risks (Tables 2 and 4). For the first time, rs4705342 were associated with asthma severity (Table 5). Moreover, the genotype–phenotype correlation analysis revealed that the rs4705342 C allele is correlated with a lower expression of miR-145-5p in serum from the healthy controls (Fig. 1A). Our studies demonstrated that the *miR-145* rs4705342 genotypes can serve as not only a novel predictor for asthma risk, but also a biomarker of asthma severe symptoms. Although the detailed mechanisms of how *miR-145* genotypes contribute to the severe symptoms remain elusive, this association may assist in predicting the prognosis of asthma patients for more precise therapy.

The detailed signaling network between miR-145-5p and asthma etiology remains unclear, but there are some clues. In 2015, Liu and colleagues reported that miR-145-5p up-regulation in airway smooth muscle cells can inhibit KLF4, and affect downstream expressions of p21, MMP-2 and MMP-9^20^. In 2017, miR-145-5p and other 4 miRNAs were confirmed to modulate the Th1/Th2 balance in asthma via regulating the expression level of Runx3 in a combinational manner^32^. In 2019, Xiong and colleagues found that miR-145-5p was up-regulated in airway epithelial cells of asthmatic mice and an miR-145-5p antagonist can significantly improve the asthmatic symptoms^34^. MiR-145-5p can promote the HDM-induced release of cytokines and epithelial barrier dysfunction via KIF3A^34^. MiR-145-5p-induced signaling pathways are complex and warrant more investigations. We provided evidence that the variant genotypes and alleles were associated with lower expression of miR-145-5p (Fig. 1A), consistent with a previous report that the presence of the C allele of rs4705342 would attenuate miR-145-5p transcription ability^22^. Since miR-145-5p is up-regulated in asthma patient^32^ and is a biomarker of reduced lung function, COPD, asthma, and other respiratory diseases^17^, it follows that lower expression of miR-145-5p would reduce the risk of asthma. In this regard, our observation that the variant genotypes of rs4705342 are correlated with lower serum levels of miR-145-5p is consistent with the observed protective effect of the variant genotypes on asthma risk.

Rs4705342 is located in the intron region of a long non-coding RNA (CARMN) and the promoter region of miR-143/miR-145 cluster. Previous studies and our own data have shown it correlated with miR-145-5p expression. GTEx did not find any expression quantitative trait locus (eQTL). Phenoscanner and REALGAR database did not find any significant correlations either, suggesting that this SNP mainly affects miR-145-5p expression, not other transcripts. Bios Consortium database found a significant methylation quantitative trait loci (meQTL) (cg03543120, *p* = 1.35 × 10^–8^), and GoDMC query found five highly significant meQTLa for rs4705342 (all *p* < 10^–77^) (cg00226225, cg04317047, cg03370704, cg03543120, and cg09660867). All these CpG sites are cis-meQTL, suggesting rs4705342 may induce differential DNA methylation on neighboring CpG sites thereby regulate miR-145-5p expression.

**Figure 2:**
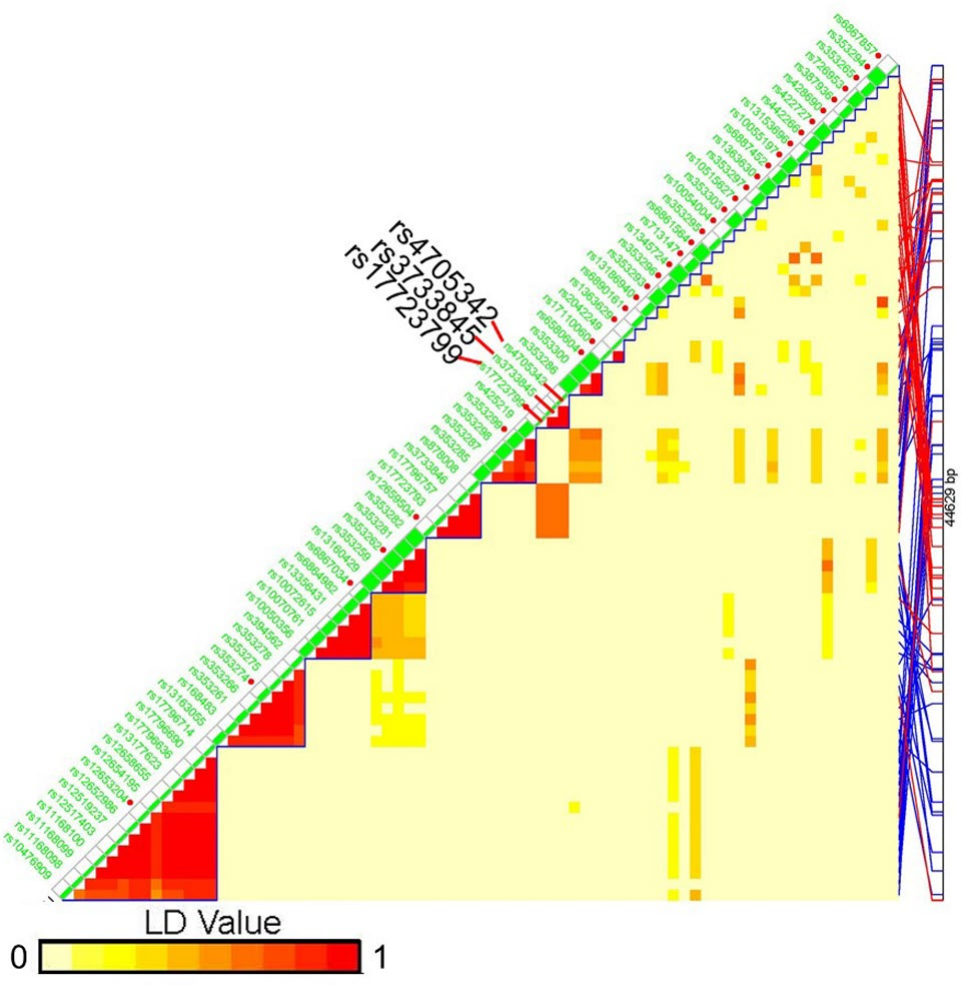
Haplotype block structure of *miR-145* genomic region. Three SNPs surrounding rs4705342 are in high linkage disequilibrium (LD) and forms a small block. Rs3733845 was genotyped in GABRIEL study and serves as a tag SNP for rs4705342.

It would be important to validate our observation in other populations. We have downloaded the summery statistics of genome-wide association study (GWAS) data of FinnGen Study and GABRIEL and queried the association of *miR-145* rs4705342 with asthma risk in these populations of European descent. The FinnGen dataset has 224,737 genotyped and phenotyped participants (including over 20,000 asthma patients)^42^. The genotype dataset included 16,383,262 SNPs (genotyped and inputted). Rs4705342 was included in the dataset and the C allele frequency in asthma cases is 18.94%, and in controls 19.06% (OR = 0.97, 95% CI 0.94–1.01, *p* = 0.11). This association was in the same direction as ours, although the effect size was much smaller. The GABRIEL study is a large-scale, consortium-based GWAS of asthma^43^. The dataset contains 582,892 SNPs in 10,365 cases and 16,110 controls from 36 studies. Rs4705342 was not genotyped and not included in the dataset. However, we found a tag SNP (rs3733845) of rs4705342 that was in the dataset. These two SNPs and another SNP (rs17723799) are in high linkage disequilibrium (LD) and form a small haplotype block (Fig. 2). *MiR-145* rs3733845 was not associated with asthma risk in Taiwan population (OR = 1.02, 95% CI 0.96–1.08, *p* = 0.54). It should be pointed out that the C allele frequency of rs4705342 is quite different across different ethnic groups (Table 6). East Asians have by far the highest frequency (more than double other ethnic groups). Different ethnic groups often have both unique and common genetic susceptibility loci. It is not surprising that rs4705342 is strongly associated with asthma risk in Taiwanese but not in other ethnic groups. Nevertheless, further validation in independent populations are warranted to confirm the association of *miR-145* rs4705342 with the risk of asthma in East Asians and Taiwanese.

**Table 6:** Minor allele (C allele) frequencies of rs4705342 and rs4705343 in different ethnic groups.

SNP	Population	Sample size	Minor allele frequency
rs4705342	European	16,114	0.073
	Estonian	4480	0.154
	African	6616	0.015
	Latin American	558	0.016
	East Asian	1008	0.319
	Japanese	16,760	0.282
	Korean	2930	0.310
	South Asian	978	0.056
rs4705343	European	75,878	0.184
	Estonian	4480	0.204
	African	41,980	0.069
	Latin American	616	0.114
	East Asian	3136	0.319
	Japanese	16,760	0.295
	Korean	2930	0.315
	South Asian	978	0.102

Data were extracted from https://www.ncbi.nlm.nih.gov/snp/rs4705342 and https://www.ncbi.nlm.nih.gov/snp/rs4705343.

## Conclusion

In conclusion, this study provides evidence for the first time that the genotypes at *miR-145* rs4705342 may serve as a predictor of asthma risk and symptom severity. There is an obvious genotype–phenotype correlation between rs4705342 genotypes and the serum levels of miR-145-5p. MiR-145-5p is a promising target of asthma, and may facilitate the prediction of asthma occurrence and severity (Supplementary information S1).

## Supplementary Information

Supplementary Information.

## Data Availability

The genotyping datasets used or analyzed during the current study are available in supplementary data. Other personalized information is available from the corresponding author on reasonable request.

## Abbreviations

miRNAs: MicroRNAs
HDM: House dust mite
Th2: Type 2 helper T cell
ORs: Odds ratios
CIs: Confidence intervals
FEV1: Forced expiratory volume in the first second
FVC: Forced vital capacity
